# Global, regional, and national analysis of CNS cancers burden from 1990 to 2021 and forecast of disease trend over the next 25 years

**DOI:** 10.1097/MD.0000000000050073

**Published:** 2026-08-14

**Authors:** Jing Wang, Lei Zhang, Yanjun Wang, Jie Liu, Peijun Han, Yuchen Guo, Yinfu Xu

**Affiliations:** a Department of Oncology, The Second People’s Hospital of Liaocheng, Liaocheng, Shandong, China; b Department of Neurosurgery, The Second People’s Hospital of Liaocheng, Liaocheng, Shandong, China.

**Keywords:** age-standardized rate, brain and central nervous system cancer, DALYs, deaths, incidence, prevalence

## Abstract

Central nervous system (CNS) cancers are highly lethal and resource-intensive, imposing a substantial and uneven burden worldwide. Comparable long-term estimates of disease burden and future trends are needed to inform prevention strategies and health system planning across regions with different socioeconomic contexts. Data were obtained from the Global Burden of Disease (GBD) 2021 study covering 204 countries and territories from 1990 to 2021. We estimated incidence, prevalence, mortality, and disability-adjusted life years (DALYs) at global, regional, and national levels. Age-standardized rates were calculated using the GBD standard population to enable comparisons across populations with different age structures. Socio-demographic Index (SDI)–based analyses and inequality metrics were applied to assess socioeconomic disparities in disease burden. Decomposition analysis was used to quantify the contributions of population growth, population aging, and changes in age-specific rates to DALY trends. Bayesian age–period–cohort (BAPC) models were employed to project future burden under the assumption that historical trends continue. In 2021, CNS cancer accounted for 0.36 million (95% UI: 0.31–0.41 million) new cases and 0.26 million (0.22–0.30 million) deaths worldwide, resulting in 8.91 million (7.61–10.36 million) DALYs. From 1990 to 2021, the global age-standardized incidence rate increased from 3.75 to 4.28 per 1,00,000 population, whereas the age-standardized DALY rate declined by 11.97%, indicating improved survival and disease management despite rising case numbers. Global prevalence increased from 4,34,000 in 1990 to 9,75,000 in 2021, reflecting the growing cumulative burden of individuals living with CNS cancer. Marked socioeconomic gradients were observed: high-SDI regions generally showed higher incidence rates but lower DALY rates, whereas low- and low-middle-SDI regions bore a disproportionate share of disability and premature mortality. Decomposition analysis indicated that population aging and population growth were the primary drivers of increasing DALYs, partially offset by reductions in age-specific rates. The global burden of CNS cancer is increasing, with marked socioeconomic disparities. These results are descriptive and scenario-based and should be interpreted in light of the assumptions and limitations of the GBD framework.

## 1. Introduction

Primary tumors of the brain and central nervous system (CNS), collectively referred to as CNS cancer, affect individuals across all age groups and can arise in various CNS anatomical regions.^[1,2]^ Despite accounting for a relatively small proportion of all cancers, CNS cancer contributes disproportionately to years of life lost and healthcare costs due to its poor prognosis, complex management, and long-term care needs among survivors.^[3]^

Over recent decades, the global burden of CNS cancer has increased, driven by population growth, population aging, and advances in diagnostic capacity. Previous studies have reported rising incidence and prevalence in many regions, alongside persistent geographic and socioeconomic disparities in outcomes. However, trends in mortality and disability-adjusted life years (DALYs) have varied substantially across countries and development levels, suggesting differences in access to timely diagnosis, treatment, and supportive care.^[4,5]^

Socioeconomic development plays a critical role in shaping the burden of CNS cancer. The Socio-demographic Index (SDI), which integrates income per capita, educational attainment, and fertility rates, provides a useful framework for examining disparities in disease burden across countries at different stages of development.^[6-8]^ Understanding how the CNS cancer burden varies by SDI level may help identify inequities in healthcare access and inform resource allocation. Beyond describing overall trends, it is important to disentangle the underlying drivers of changes in the CNS cancer burden. Decomposition analysis enables the separation of the contributions of population growth, population aging, and changes in age-specific rates to DALY trends, thereby distinguishing demographic from epidemiological influences. In addition, frontier analysis allows benchmarking of observed disease burden against the lowest DALY rates achieved at comparable levels of socioeconomic development, providing insight into potentially achievable reductions under optimal conditions.^[9,10]^

Using data from the Global Burden of Disease (GBD) 2021 study, this study aimed to provide a comprehensive assessment of the global burden of CNS cancer from 1990 to 2021. Specifically, we sought to characterize long-term temporal trends in incidence, prevalence, mortality, and disability-adjusted life years using age-standardized rates (ASR) and estimated annual percentage changes; to examine socioeconomic inequalities in the CNS cancer burden across regions and levels of socio-demographic development; and to project future burden under scenario-based assumptions using Bayesian age–period–cohort models, with the ultimate goal of informing long-term health system planning and resource allocation.

## 2. Materials and methods

### 2.1. Data sources

The GBD 2021 study builds upon its predecessors by providing an expanded analysis of epidemiological data across 204 countries, 21 geographic regions, 369 health conditions, and 87 risk factors. This comprehensive evaluation encompasses incidence, prevalence, mortality, and DALYs.^[11]^ A multinational team of researchers compiled the data from published studies, surveys, and health records, ensuring a robust and comprehensive dataset.^[12-14]^

Countries were categorized into 5 groups based on their SDI: low, lower-middle, middle, upper-middle, and high SDI. The SDI serves as a composite measure to annually evaluate the developmental status of each region, incorporating 3 key metrics: income per capita, educational attainment among individuals aged 15 and older, and fertility rates among women under 25. An SDI score of 0 represents the theoretical minimum for health-related development, while a score of 1 signifies the maximum.^[15-17]^

For this analysis, the world was divided into 21 regions. Data on CNS cancer incidence, prevalence, DALYs, and corresponding age-standardized rates (ASRs) were retrieved using the Global Health Data Exchange query tool (http://ghdx.healthdata.org/gbd-results-tool). ASRs were calculated based on the GBD World Population Criteria, and all estimates were reported with a 95% uncertainty interval (UI).^[18]^

### 2.2. CNS cancer definition

The classification of CNS cancers adheres to the diagnostic guidelines outlined in the International Classification of Diseases (ICD). Within the ICD-11 framework, CNS cancers are categorized under codes C70.0–C72.9. Specifically, code C70 refers to malignant neoplasms of the meninges, C71 to malignant brain tumors, and C72 to malignant neoplasms of the spinal cord, cranial nerves, and other parts of the CNS.^[19]^

### 2.3. Statistical analyses

This study utilized ASR for incidence, prevalence, mortality, DALYs, and estimated annual percentage changes (EAPCs) in prevalence to quantify the global impact of CNS cancers. Standardization is essential for comparing populations with varying age distributions and for tracking temporal changes within a population. ASR, expressed per 1,00,000 individuals, provides insights into shifts in disease patterns and potential changes in risk factors, enabling the development of targeted prevention and treatment strategies. ASRs were calculated using the GBD standard population to ensure comparability across countries and over time. EAPCs were derived by fitting a linear regression model to the natural logarithm of the ASR over the period from 1990 to 2021. EAPC serves as a comprehensive metric to analyze ASR trends over specified intervals. The regression model is based on the natural logarithm equation: *y* = α + β*x* + ε, where y represents ln(ASR), x denotes the calendar year, and ε signifies the error term.^[20]^ EAPC is reported with a 95% confidence interval (CI). An upward trend in ASR is identified when both the EAPC estimate and its lower 95% CI exceed zero, while a downward trend is observed if both the EAPC estimate and its upper 95% CI are below zero.^[21]^ Otherwise, ASR is considered stable over time. Pearson correlation coefficient was used to evaluate the relationship between ASR and SDI regions.

Decomposition analysis was employed to identify factors contributing to changes in DALYs from 1990 to 2021. Overall variations in DALYs were attributed to 3 components: changes in age structure, population growth, and epidemiological transitions reflected in age-specific DALY rates. Counterfactual scenarios were constructed by sequentially holding 2 components constant while allowing the third component to vary, following the standard GBD decomposition framework. Changes in DALYs were thus attributed to population growth, population aging, and changes in age-specific rates under these counterfactual assumptions. Because each component was estimated relative to different counterfactual baselines, the summed percentage contributions may exceed 100% due to interaction effects among components. These percentages should therefore be interpreted as directional contributions rather than exact proportional shares of change. To project the future global burden of CNS cancer, Bayesian age-period-cohort (BAPC) modeling was used to estimate disease trends for the next 25 years.^[22]^ BAPC models were implemented using standard specifications as described in previous GBD-related studies. Briefly, age, period, and cohort effects were modeled using second-order random walk priors to achieve temporal smoothing. Weakly informative priors were applied to all model parameters, and no manual tuning of prior distributions was performed. Model projections are scenario-based and assume the continuation of historical trends rather than providing deterministic forecasts. Formal out-of-sample validation and additional model diagnostics were not conducted, consistent with the descriptive and exploratory aim of long-term burden projection in this study. The concentration index reflects the extent to which disease burden is disproportionately distributed across the socioeconomic spectrum. A positive concentration index indicates that the burden is concentrated in countries with higher SDI, whereas a negative value indicates concentration in lower-SDI settings. The slope index of inequality represents the absolute difference in disease burden between the highest and lowest ends of the SDI spectrum, with larger absolute values indicating greater inequality. Changes in these indices over time reflect shifts in the magnitude and direction of socioeconomic disparities rather than changes in overall disease burden. Statistical analyses and visualizations were performed using R software (version 4.4.2, RStudio) and GraphPad Prism software (version 9.3.1; GraphPad Software, LLC). Statistical significance was defined as a *P*-value of <.05.

## 3. Results

### 3.1. Global trends in CNS cancer burden from 1990 to 2021

From 1990 to 2021, the global burden of CNS cancer showed divergent trends across key metrics. While the age-standardized incidence rate increased steadily over the study period, the age-standardized DALY rate declined, indicating improvements in survival and disease management despite a growing number of new cases. In contrast, absolute counts of incident cases, deaths, and DALYs increased substantially, largely reflecting population growth and population aging.

This divergence between rising incidence and declining DALY rates was consistently observed across multiple regions and was further supported by decomposition analyses, which showed that demographic factors were the primary drivers of increases in total DALYs, partially offset by reductions in age-specific rates. Detailed numerical estimates of global counts and rates are presented in Table 1 and Supplementary Tables for reference.

**Table 1:** The incidence cases and age-standardized incidence of CNS cancer in 1990 and 2021, and its temporal trends from 1990 to 2021.

Characteristics	1990		2021		1990–2021
	Incidence cases No .×10’5 (95% UI)	ASR per 1,00,000 No. (95% UI)	Incidence cases No. ×10’5 (95% UI)	ASR per 1,00,000 No. (95% UI)	EAPC No. (95% CI)
Global	1,73,086.44 (1,47,452.47, 1,94,951.22)	3.75 (3.21, 4.21)	3,57,482.30 (3,10,456.68, 4,07,432.57)	4.28 (3.71, 4.88)	1.112 (1.085, 1.138)
High SDI	55,694.12 (54,173.05, 56,847.27)	5.66 (5.52, 5.78)	1,00,993.68 (94,963.67, 1,05,628.62)	6.38 (6.08, 6.64)	1.247 (1.185, 1.309)
High-middle SDI	49,615.13 (42,241.78, 55,310.85)	4.81 (4.10, 5.35)	97,818.17 (83,423.12, 1,12,504.75)	5.90 (5.05, 6.77)	1.588 (1.561, 1.616)
Middle SDI	47,057.61 (35,595.13, 56,516.65)	3.35 (2.57, 4.07)	1,06,918.22 (86,956.26, 1,28,833.12)	4.11 (3.34, 4.94)	1.547 (1.497, 1.596)
Low-middle SDI	15,846.92 (11,647.73, 21,805.63)	1.73 (1.31, 2.32)	39,604.13 (31,857.51, 49,413.71)	2.34 (1.89, 2.93)	1.415 (1.396, 1.434)
Low SDI	4669.47 (2949.14, 7298.29)	1.23 (0.77, 1.75)	11,810.39 (8189.76, 15,144.36)	1.43 (1.00, 1.82)	0.412 (0.286, 0.539)
Andean Latin America	838.33 (665.07, 1161.40)	2.76 (2.20, 3.73)	2793.67 (2221.97, 3479.50)	4.43 (3.52, 5.50)	2.358 (2.053, 2.664)
Australasia	1393.20 (1343.26, 1447.54)	6.32 (6.10, 6.56)	2536.34 (2341.92, 2729.75)	5.95 (5.54, 6.38)	0.620 (0.535, 0.705)
Caribbean	896.55 (823.71, 1130.73)	2.93 (2.72, 3.61)	1971.17 (1725.14, 2269.86)	3.85 (3.37, 4.46)	1.969 (1.856, 2.083)
Central Asia	1965.95 (1639.67, 2244.45)	3.24 (2.68, 3.69)	4635.68 (4012.80, 5300.49)	4.94 (4.29, 5.62)	1.931 (1.797, 2.064)
Central Europe	8003.42 (7657.03, 8532.38)	5.76 (5.50, 6.15)	11720.65 (10,689.02, 12,787.13)	6.64 (6.07, 7.25)	1.623 (1.386, 1.862)
Central Latin America	2825.21 (2739.98, 2916.07)	2.23 (2.17, 2.29)	7585.80 (6775.25, 8533.38)	2.98 (2.66, 3.36)	1.626 (1.416, 1.838)
Central Sub-Saharan Africa	294.70 (213.23, 419.46)	0.83 (0.60, 1.07)	876.40 (586.26, 1181.94)	1.03 (0.67, 1.39)	0.689 (0.496, 0.881)
East Asia	48,577.77 (35,371.79, 60,360.49)	4.63 (3.39, 5.77)	1,07,613.78 (83,225.28, 1,35,767.79)	6.02 (4.69, 7.53)	1.998 (1.927, 2.068)
Eastern Europe	9101.42 (8399.80, 9678.27)	3.63 (3.37, 3.85)	14,293.12 (13,133.92, 15,494.25)	4.98 (4.60, 5.38)	1.702 (1.596, 1.809)
Eastern Sub-Saharan Africa	1847.68 (1233.90, 2724.86)	1.25 (0.81, 1.67)	4820.69 (3456.72, 6278.22)	1.53 (1.05, 1.92)	0.621 (0.499, 0.744)
High-income Asia Pacific	5648.89 (5036.86, 5978.06)	3.11 (2.76, 3.29)	16,367.65 (14,108.19, 18,000.79)	5.44 (4.76, 5.92)	3.456 (3.137, 3.775)
High-income North America	22,169.59 (21,557.53, 22,637.46)	7.11 (6.93, 7.24)	36,461.77 (34,376.30, 37,685.94)	7.08 (6.75, 7.31)	0.736 (0.663, 0.809)
North Africa and Middle East	10,151.00 (7655.94, 14,314.71)	4.09 (3.16, 5.72)	26,565.65 (19,733.69, 32,478.58)	4.98 (3.70, 6.10)	1.320 (1.229, 1.412)
Oceania	32.44 (16.14, 43.49)	0.69 (0.35, 0.94)	82.43 (42.76, 109.56)	0.74 (0.38, 0.99)	0.581 (0.503, 0.658)
South Asia	13,984.85 (9077.78, 18,699.09)	1.61 (1.05, 2.12)	31,817.26 (26,008.92, 43,157.13)	1.87 (1.53, 2.55)	0.836 (0.705, 0.967)
Southeast Asia	7050.98 (5029.09, 8698.73)	1.97 (1.43, 2.45)	16,690.80 (12,169.79, 19,949.90)	2.40 (1.76, 2.87)	1.504 (1.444, 1.564)
Southern Latin America	1485.49 (1349.82, 1622.37)	3.12 (2.84, 3.41)	2981.41 (2776.22, 3172.72)	3.81 (3.55, 4.06)	1.738 (1.452, 2.026)
Southern Sub-Saharan Africa	569.10 (441.13, 728.86)	1.57 (1.20, 1.98)	1390.09 (1024.05, 1668.24)	2.05 (1.50, 2.43)	1.568 (1.485, 1.652)
Tropical Latin America	4966.58 (4716.16, 5251.44)	4.14 (3.93, 4.40)	14,100.91 (13,433.54, 14,672.84)	5.65 (5.37, 5.88)	2.172 (1.978, 2.366)
Western Europe	30,521.26 (29,739.83, 31,118.08)	6.55 (6.40, 6.66)	49,619.44 (46,606.04, 51,778.61)	7.44 (7.13, 7.71)	1.226 (1.109, 1.343)
Western Sub-Saharan Africa	762.05 (514.84, 997.27)	0.43 (0.30, 0.53)	2557.60 (1314.70, 3276.16)	0.63 (0.34, 0.79)	1.159 (1.020, 1.298)

ASR = age-standardized rate, CI = confidence interval, CNS = central nervous system, EAPC = estimated annual percentage change, SDI = Socio-demographic Index, UI = uncertainty interval.

**Figure 1.**
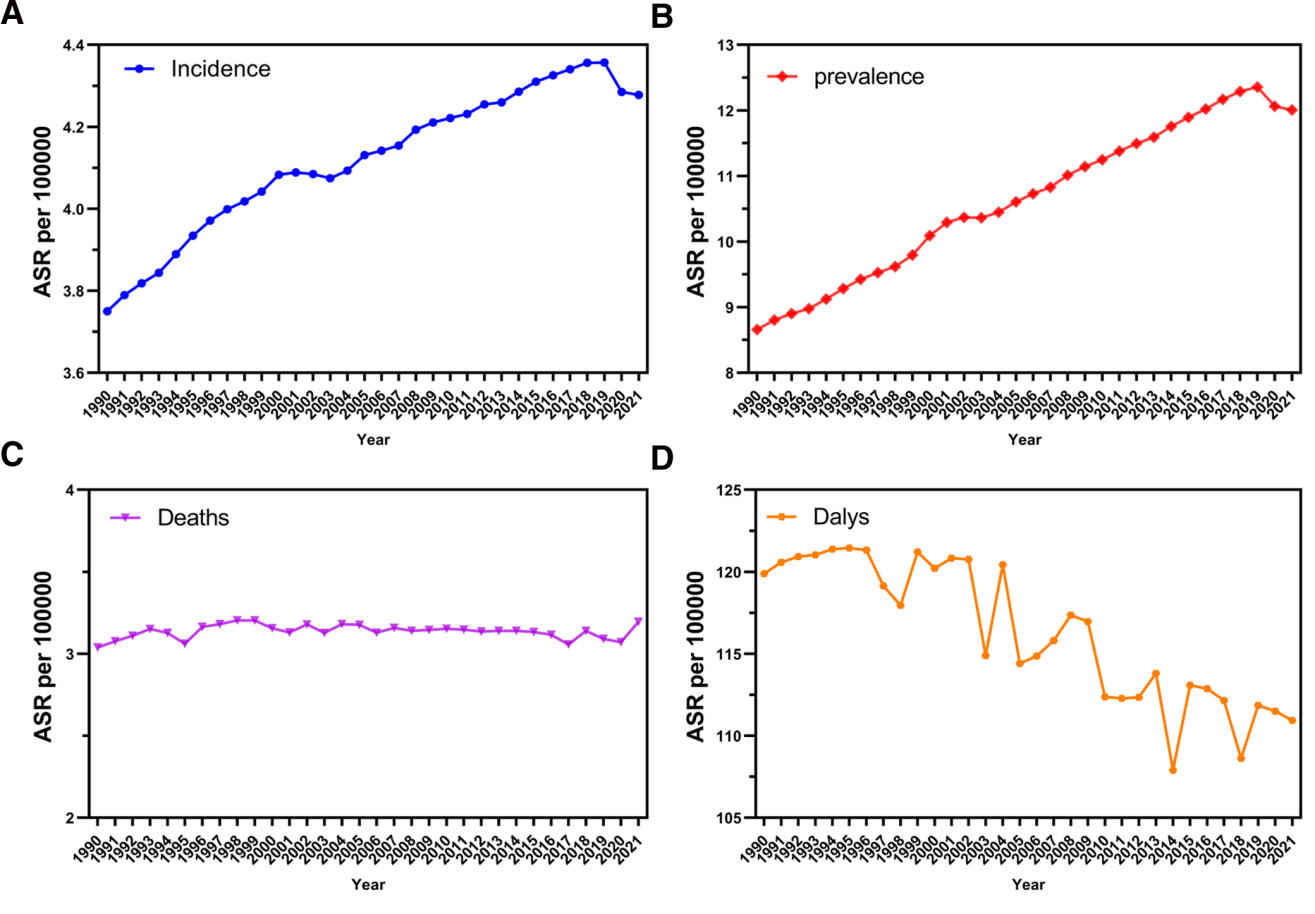
Age-standardized incidence rates, prevalence, deaths, and DALY change curves for CNS cancer patients from 1990 to 2021. (A: Incidence; B: Prevalence; C: Deaths; D: DALYs). CNS = central nervous system, DALYs = disability-adjusted life years.

Over the study period, a distinct trend emerged: while age-standardized rates for incidence and prevalence gradually increased, the ASDR consistently declined, and the age-standardized mortality rate approached stabilization (Fig. 1).

### 3.2. Worldwide age profile of CNS cancer burden in 2021

**Figure 2.**
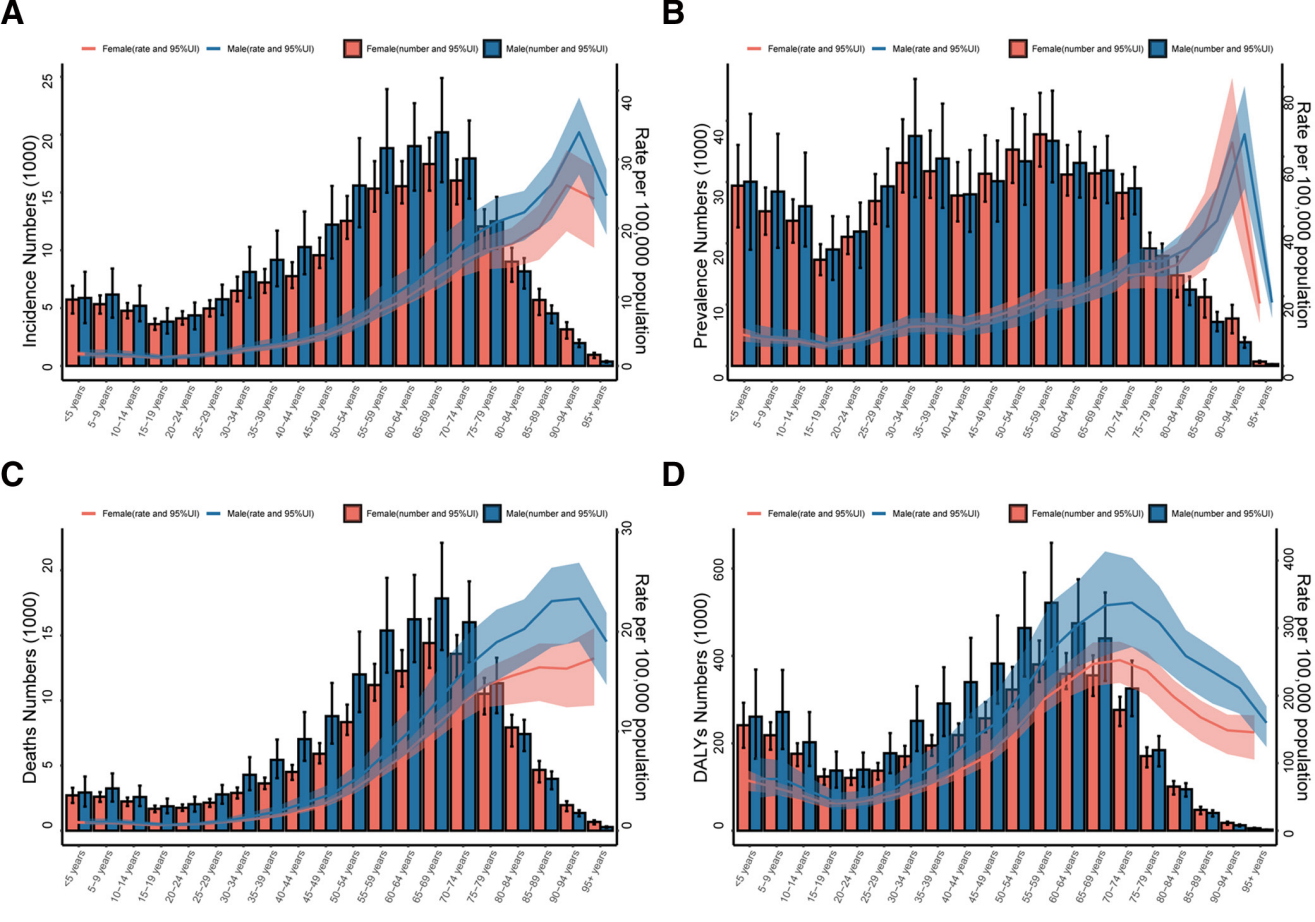
The number of CNS cancer patients of each age group (per 5-year cohort) in males and females with incidence, prevalence, death, and DALYs and their ASR, with the number on the left side and the ASR on the right side, with females in pink and males in blue. (A: Incidence; B: Prevalence; C: Deaths; D: DALYs). ASR = age-standardized rate, CNS = central nervous system, DALYs = disability-adjusted life years.

The global prevalence, incidence, mortality, and DALY rates for CNS cancer indicated a consistent upward trend with advancing age (Fig. 2 and Table S1, Supplemental Digital Content 1). These rates significantly increased across age groups, peaking among individuals aged 90 to 94 years, followed by a gradual decline after 95 years of age (Fig. 2A–C). In contrast, the DALY rate increased steadily with age, peaking in the 70 to 74 age group before gradually declining, particularly after age 74 (Fig. 2D).

### 3.3. Sex-based distribution of global CNS cancer burden

From 1990 to 2021, both genders experienced increases in CNS cancer incidence, prevalence, mortality, DALYs, and their respective rates. Overall, the disease burden was slightly higher in males than in females (Fig. S1, Supplemental Digital Content 2). However, in 2021, notable gender disparities emerged across age groups. Females exhibited higher numbers of new cases, deaths, and DALYs at age 80 and greater overall prevalence after age 75 (Fig. 2). According to Tables S2 and S3, Supplemental Digital Content 3, the 2021 incidence rate of CNS cancer was 3.9 per 1,00,000 (95% UI: 3.4–4.3) for females and 4.7 per 1,00,000 (95% UI: 3.7–5.8) for males. The prevalence rates were 11.8 per 1,00,000 (95% UI: 10.5–13.3) for females and 12.3 per 1,00,000 (95% UI: 9.7–15.0) for males. Mortality rates were 2.6 per 1,00,000 (95% UI: 2.3–2.9) for females and 3.5 per 1,00,000 (95% UI: 2.7–4.3) for males. The DALY rate was 93.5 per 1,00,000 (95% UI: 82.9–104.1) for females and 123.0 per 1,00,000 (95% UI: 93.6–157.3) for males.

Overall, males exhibited higher incidence and DALY rates of CNS cancer than females across most age groups. However, in older age groups, particularly among individuals aged ≥ 75 years, females showed comparable or higher DALY rates than males. This pattern was mainly driven by a higher burden at older ages rather than higher incidence, suggesting that sex differences in survival, population age structure, or data completeness may contribute to the observed disparities.

### 3.4. Global distribution of CNS cancer disease burden across regions and countries

**Figure 3.**
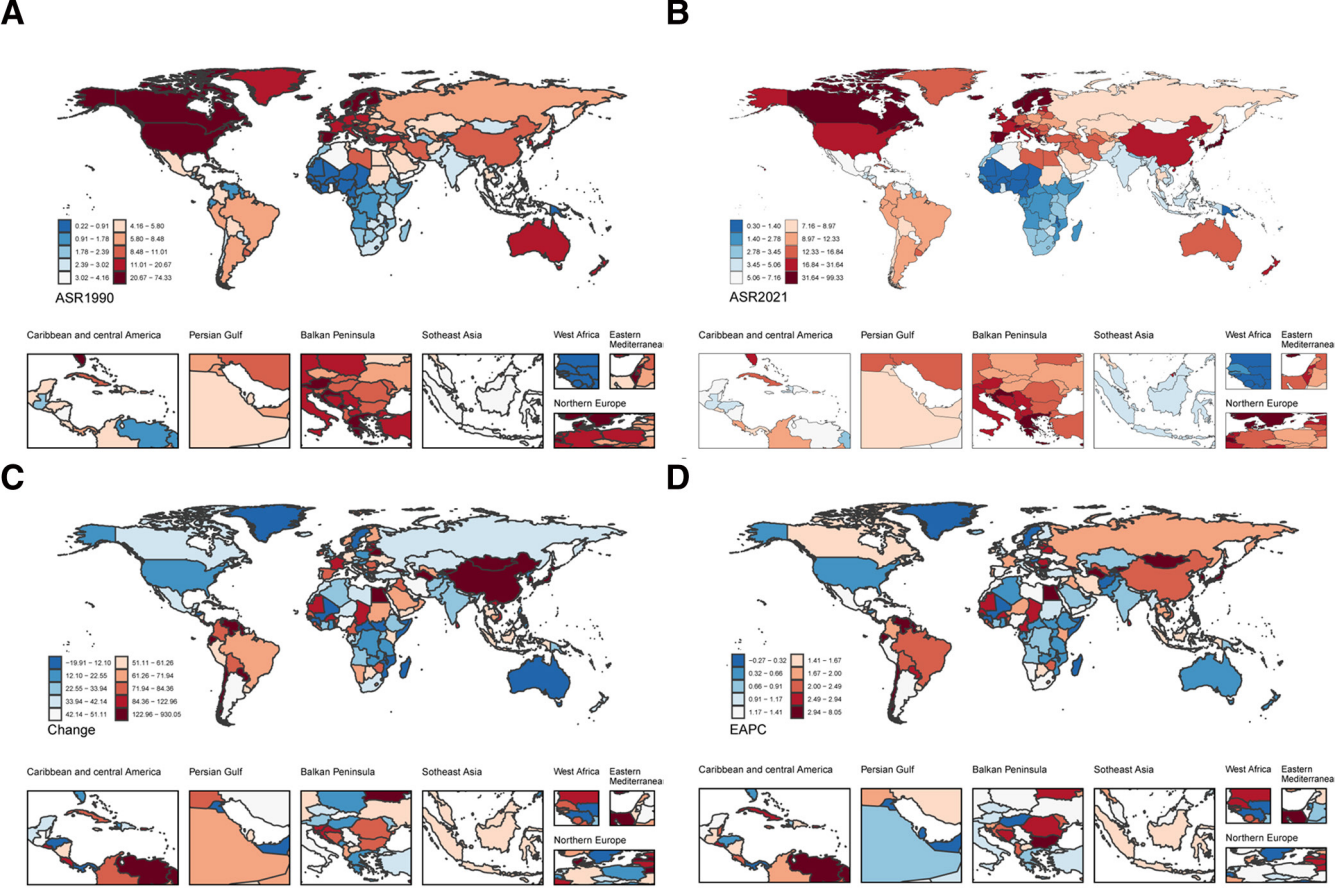
Global age-standardized prevalence of CNS cancer for both sexes in 204 countries and territories. (A) Age-standardized prevalence of CNS cancer in 1990, (B) age-standardized prevalence of CNS cancer in 2021, (C) the relative change in prevalence of CNS cancer between 1990 and 2021, and (D) the EAPC of the prevalence of CNS cancer between 1990 and 2021. CNS = central nervous system, EAPC = estimated annual percentage change.

The geographical distribution heat map (Fig. 3) highlights significant variations in the burden of CNS cancer across nations in 1990. The countries with the highest prevalence rates included China (1,08,115), the United States (55,754), Japan (24,504), India (22,567), and Italy (11,169). After adjusting for age and demographic factors, the top 5 countries by prevalence rate per 1,00,000 were San Marino (73.6), Norway (70.4), Sweden (69.2), Iceland (57.8), and Greece (54.6; Fig. 3A). By 2021, the burden of CNS cancer exhibited significant variability across countries and regions. The nations with the highest prevalence were China (3,05,063), the United States (84,402), Japan (70,806), India (49,147), and South Korea (29,142). After adjusting for age and population size, the top 5 countries by prevalence rate per 1,00,000 were Norway (98.3), Denmark (88.8), Monaco (71.7), San Marino (71.5), and Sweden (70.6; Fig. 3B).

From 1990 to 2021, the countries with the largest increases in prevalence rates were Ecuador (912.6%), Turkmenistan (467.6%), Tokelau (353.4%), South Korea (333.8%), and Kyrgyzstan (294.6%; Fig. 3C). When adjusted for age and population factors, the top 5 countries in prevalence changes, as measured by EAPC, were Ecuador (+8.0%), Turkmenistan (+7.8%), Georgia (+6.0%), Kyrgyzstan (+4.6%), and Mongolia (+4.3%; Fig. 3D). Overall, the burden of CNS cancer was generally greater in countries with limited healthcare resources.

### 3.5. Trends in CNS cancer disease burden by SDI level

**Figure 4.**
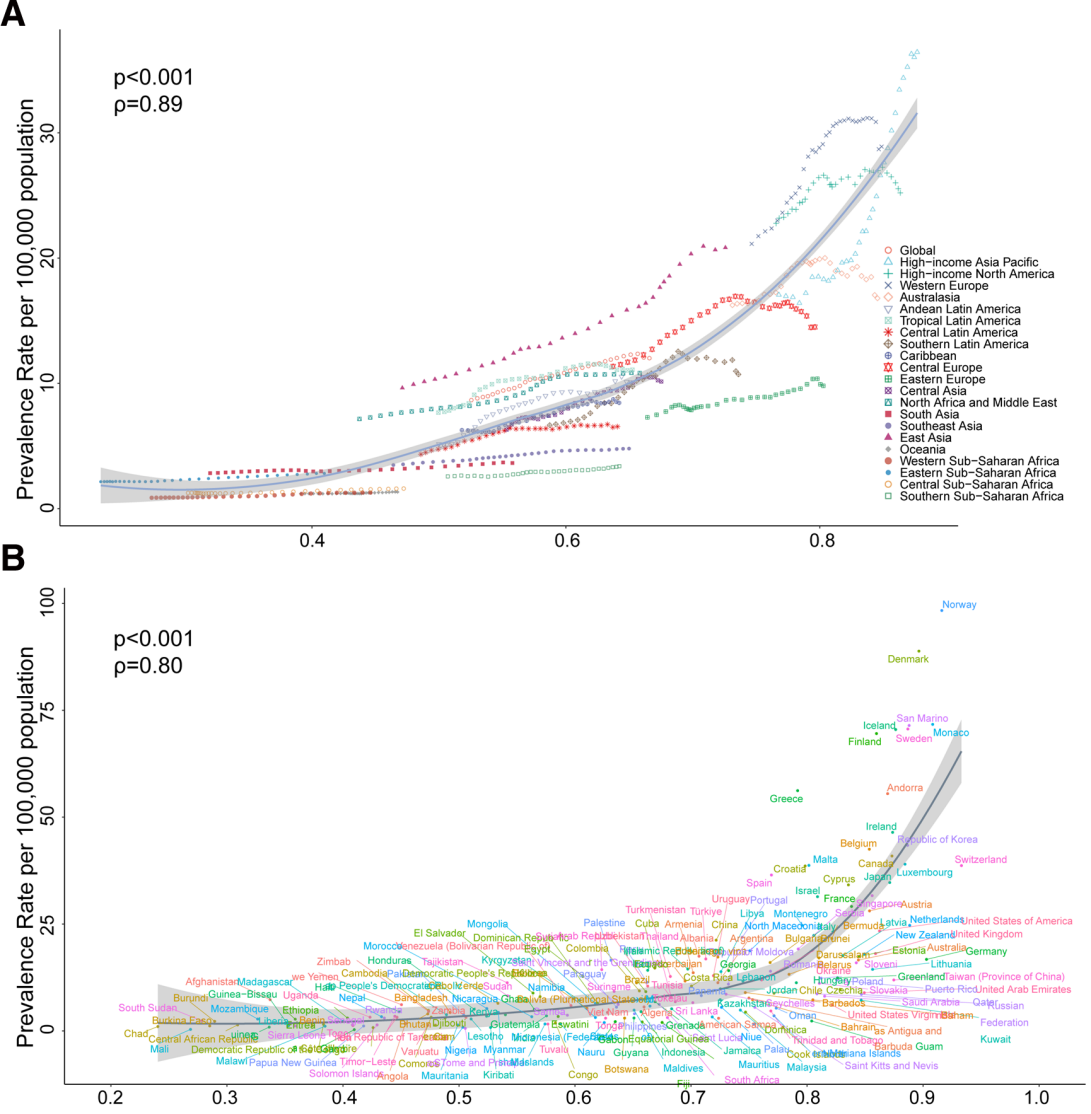
Age-standardized prevalence rates for CNS cancer for 21 GBD regions (A) and 204 countries and territories (B) by Socio-demographic Index. 1990–2021 expected values based on Socio-demographic Index and disease rates in all locations are shown as the blue line. CNS = central nervous system, DALYs = disability-adjusted life years, GBD = Global Burden of Disease.

The study analyzed 204 nations and 21 regions, grouped into 5 SDI levels, revealing distinct disparities in the evolution of the CNS cancer burden across these categories (Fig. 4). As depicted in Fig. S2, Supplemental Digital Content 4, standardized incidence, prevalence, mortality, and DALY rates were higher in high-middle and high SDI regions; however, standardized mortality and DALY rates demonstrated a declining trend. In contrast, low-middle SDI regions exhibited a more pronounced increase in standardized mortality rates and DALYs. Despite this trend, DALYs associated with CNS cancer remained significantly elevated in high-middle SDI regions. SDI patterns across the 21 GBD regions depicted considerable variability. Some regions experienced minimal changes in ASR over the study period, while others displayed fluctuating or rising trends, with a correlation coefficient of ρ = 0.89, *P* < .001 (Fig. 4A). Globally, ASR for 204 countries and territories in 2021 consistently increased with higher SDI levels, reflected by a correlation coefficient of ρ = 0.80, *P* < .001 (Fig. 4B). In several low-SDI settings, relatively low recorded incidence coexisted with disproportionately high DALY rates, reflecting a pattern of severe disability and premature mortality rather than a low disease burden.

### 3.6. Decomposition analysis in DALYs

The findings revealed that population growth from 1990 to 2021 accounted for 96.68% of the increased DALY burden from CNS cancer, followed by demographic aging (31.35%) and epidemiological transitions (–28.04%). Aging had the greatest impact on total DALYs in the low SDI quintile (44.26%), with subsequent declines observed in the middle (29.59%), low-middle (22.2%), high (–0.18%), and high-middle SDI quintiles (–3.05%). This pattern was largely driven by significant population growth in the high-middle SDI quintile (148.37%).

During the study period, epidemiological shifts exhibited varied effects. Positive impacts were observed in the low SDI (8.63%) and low-middle SDI (21.8%) quintiles, while negative effects were recorded in the high (–27.91%), middle (–28.62%), and high-middle SDI quintiles (–45.32%; Table 2 and Fig. S3, Supplemental Digital Content 5).

**Table 2:** Changes in DALYs by population-level determinants from 1990 to 2021 globally and by sociodemographic index quintile.

Location	Overall difference	Change due to population-level determinants (contribution to the total change)		
		Aging	Population	Epidemiological change
Global	29,54,114.24	9,26,186.93 (31.35)	28,56,151.23 (96.68)	−8,28,223.92 (−28.04)
High SDI	4,68,273.49	−832.45 (-0.18)	5,99,812.3 (128.09)	−1,30,706.36 (−27.91)
High-middle SDI	5,12,809.95	−15,664.37 (-3.05)	7,60,877.22 (148.37)	−2,32,402.9 (-45.32)
Middle SDI	9,29,976.26	2,75,199.16 (29.59)	9,20,953.91 (99.03)	−2,66,176.81 (-28.62)
Low-middle SDI	7,34,563.74	1,63,044.14 (22.2)	4,11,391.03 (56)	1,60,128.56 (21.8)
Low SDI	3,06,297.28	1,35,580.82 (44.26)	1,44,278.03 (47.1)	26,438.43 (8.63)

CNS = central nervous system, DALYs = disability-adjusted life years, SDI = Socio-demographic Index.

### 3.7. Cross-country analysis of inequality in CNS cancer

**Figure 5.**
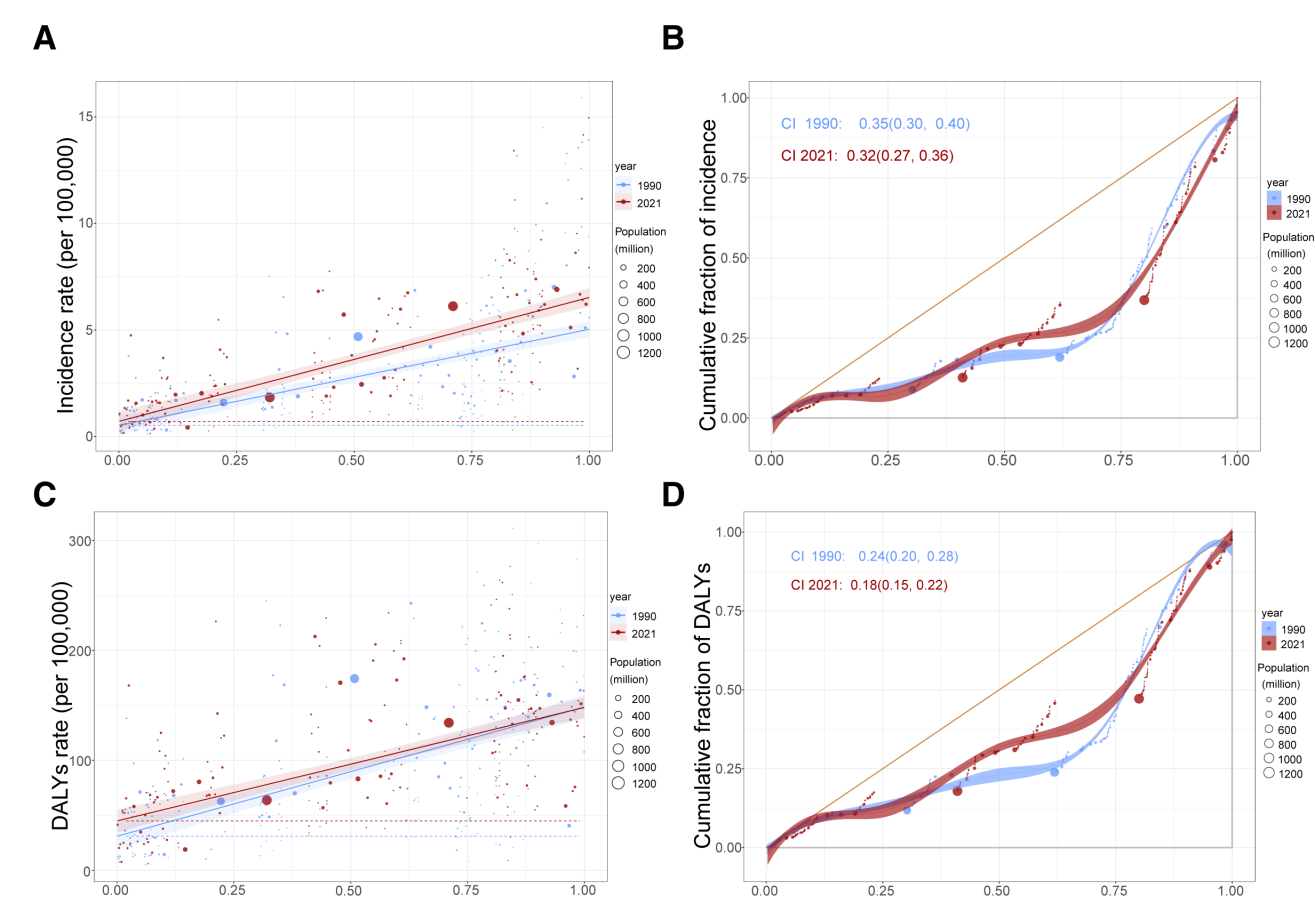
Inequality indices for CNS cancer rates: incidence and DALYs. Due to the multidimensional nature of inequality metrics, multiple indicators are displayed to illustrate overall patterns; detailed numerical values and sensitivity information are provided in the Supplementary Materials. (A) Slope index: absolute inequality in SDI-related incidence. (B) Concentration index: relative inequality in SDI-related incidence. (C) Slope index: absolute inequality in SDI-related DALYs. (D) Concentration index: relative inequality in SDI-related DALYs. CNS = central nervous system, DALYs = disability-adjusted life years, SDI = Socio-demographic Index.

We examined health inequalities in CNS cancer across nations with different SDI levels, focusing on both absolute and relative disparities in incidence, prevalence, mortality, DALYs, and their association with SDI. Our analysis shows that these disparities have grown over time. Figure 5A, B illustrate that countries with higher SDI have higher incidence rates, with the crude rate difference increasing from 4.52 (95% CI, 3.71–5.32) in 1990 to 5.82 (95% CI, 4.98–6.66) in 2021. The concentration index, though marginally reduced from 0.35 (95% CI, 0.30–0.40) to 0.32 (95% CI, 0.27–0.36), remains positive. For prevalence (Fig. S4A, B, Supplemental Digital Content 6), the crude rate difference between the highest and lowest SDI countries rose from 10.78 (95% CI, 8.87–12.69) to 17.44 (95% CI, 14.91–19.97) from 1990 to 2021. The concentration index decreased slightly from 0.52 (95% CI, 0.45–0.57) to 0.49 (95% CI, 0.43–0.54), but stayed positive. Regarding mortality (Fig. S4C, D, Supplemental Digital Content 6), the crude rate difference increased from 3.18 (95% CI, 2.58–3.78) to 3.30 (95% CI, 2.75–3.86) between the highest and lowest SDI countries. The concentration index decreased from 0.26 (95% CI, 0.22–0.30) to 0.21 (95% CI, 0.17–0.25), but continued to be positive. In terms of DALYs (Fig. 5C, D), the crude rate difference between the highest and lowest SDI countries slightly decreased from 117.57 (95% CI, 94.44–140.69) to 103.27 (95% CI, 82.82–123.72), while the concentration index declined from 0.24 (95% CI, 0.20–0.28) to 0.18 (95% CI, 0.15–0.22) but remained positive. Although the concentration indices have slightly reduced, their persistent positive values highlight the continued health inequalities, stressing the necessity for further investigation and interventions to mitigate these global disparities (Fig. 5).

### 3.8. Frontier analysis of CNS cancer and country development status

**Figure 6.**
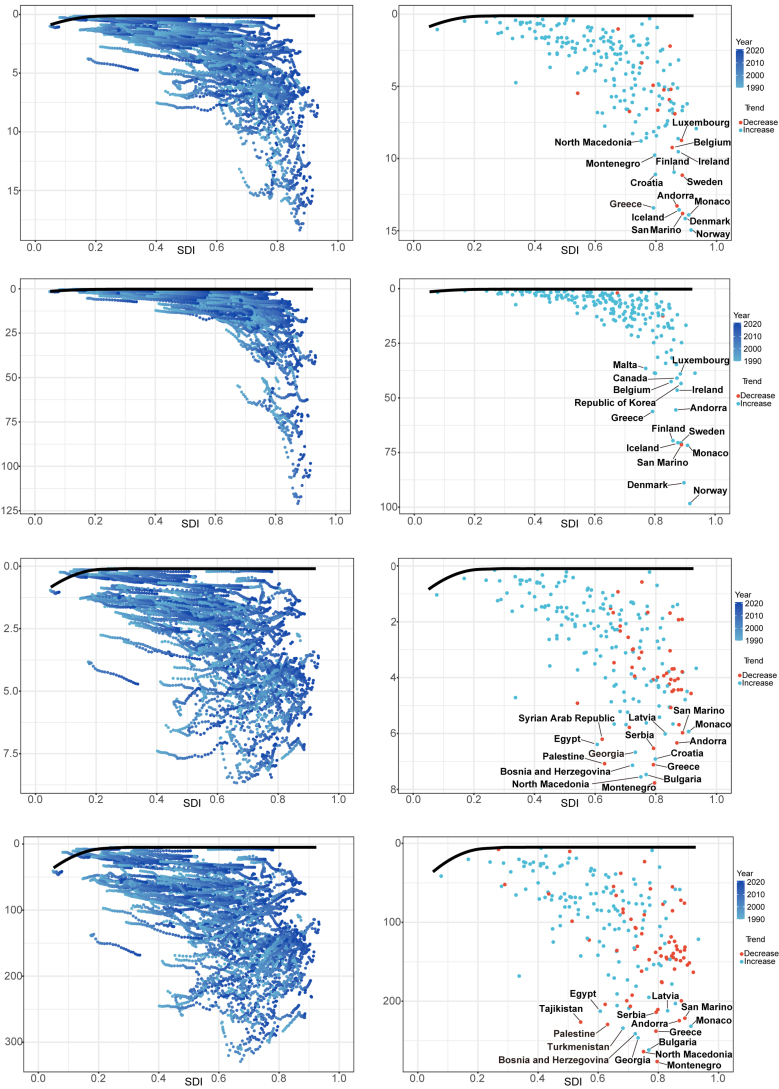
Frontier analysis of CNS cancer in 204 countries and regions in 2021, highlighting gaps in ASIR (A, B), ASPR (C, D), age-standardized mortality rate (E, F), and ASDR (G, H) between countries and the frontier. The frontier represents the lowest observed burden achieved at comparable SDI levels and serves as a descriptive benchmark for cross-country comparison. The 15 countries furthest from the frontier are marked. Blue dots indicate an increase in ASR from 1990 to 2021, while red dots denote a decrease. The figure is designed to illustrate overall frontier patterns across countries rather than to emphasize individual country positions. Given the large number of countries included, labels are shown to aid interpretation of general trends. ASDR = age-standardized DALY rate, ASIR = age-standardized incidence rate, ASPR = age-standardized prevalence rate, CNS = central nervous system, SDI = sociodemographic index.

Figure 6 presents frontier analyses of ASIR, ASPR, and ASDR across countries. These analyses show substantial cross-country heterogeneity in the relationship between SDI and observed CNS cancer burden in 2021. For each indicator, the frontier represents the lowest observed burden achieved at comparable SDI levels and serves as a descriptive benchmark rather than a causal or normative standard. Several countries, including Malta, Luxembourg, Canada, Denmark, Norway, and Monaco, were located relatively far from the frontier for selected indicators. In DALY analyses, countries such as Montenegro, North Macedonia, Bulgaria, Greece, Turkmenistan, Monaco, and Palestine also showed relatively large gaps from the frontier. These deviations should be interpreted cautiously, as they may reflect a combination of diagnostic intensity, registry completeness, survival patterns, and health system factors rather than poorer disease control alone.

### 3.9. Prediction of the global disease burden of CNS cancer

**Figure 7.**
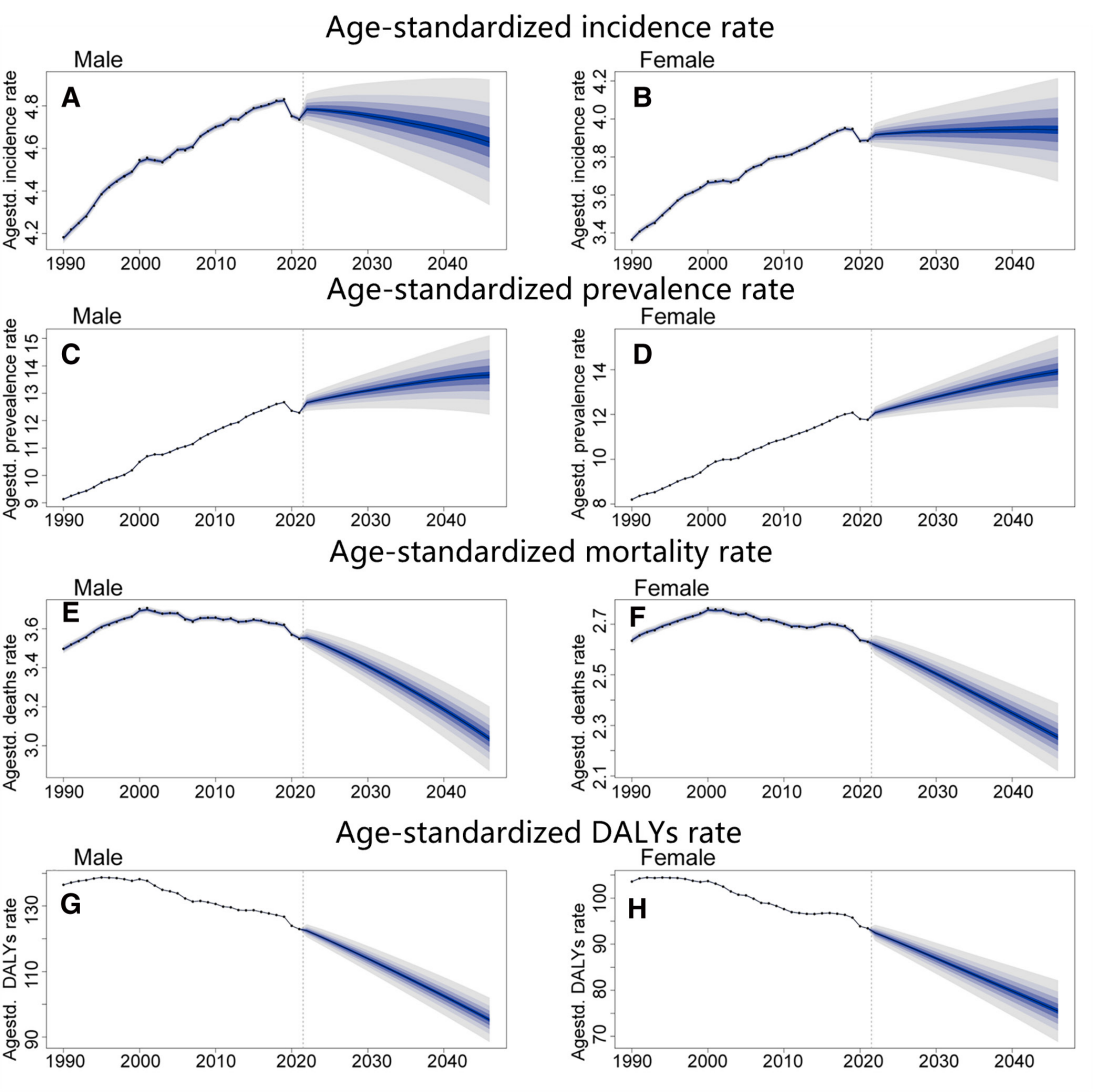
Trends in the CNS cancer age-standardized incidence, the age-standardized prevalence, age standardized mortality rate, and the age-standardized DALYs by sex in global: observed (solid lines) and predicted rates (dashed lines). The blue region shows the upper and lower limits of the 95% uncertainty intervals (95% UIs). Solid lines represent observed estimates from 1990 to 2021, while dashed lines indicate projected trends from 2022 to 2050 based on the Bayesian age–period–cohort model. Shaded bands denote the 95% uncertainty intervals, reflecting uncertainty arising from data availability, model assumptions, and parameter estimation. CNS = central nervous system, DALYs: disability-adjusted life years; UI: uncertainty interval.

**Figure 8.**
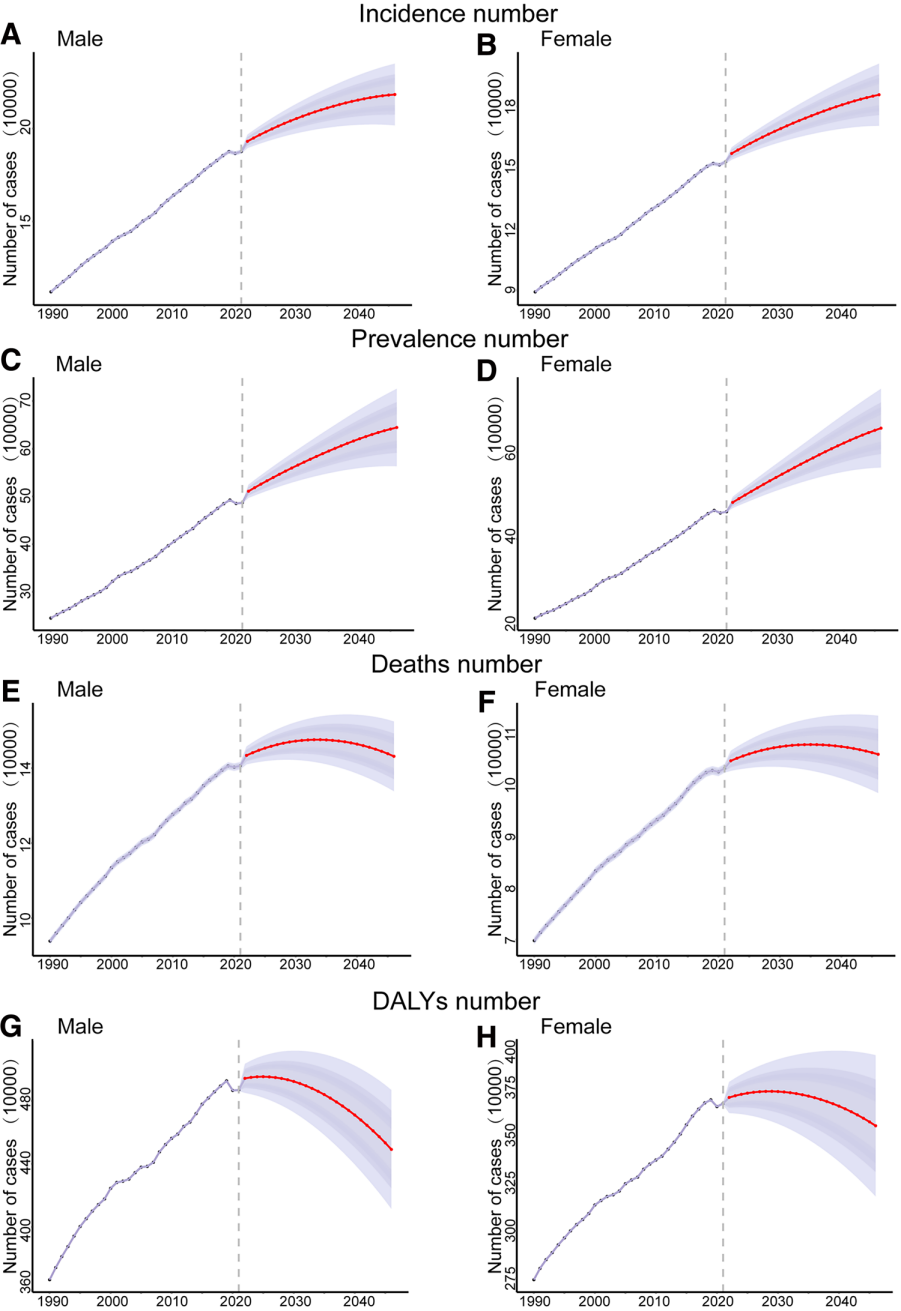
Global trends in the numbers of incident cases (A, B), prevalent cases (C, D), deaths, and DALYs (G, H) of CNS cancer by sex: observed estimates before 2021 and projected counts after 2021. Dotted curves show the baseline BAPC projections. Shaded areas indicate scenario-based ranges generated under assumptions that the corresponding rate remains stable, decreases by 1% per year, or increases by 1% per year after 2021. These shaded regions represent scenario envelopes rather than 95% uncertainty intervals. BAPC = Bayesian age–period–cohort, CNS = central nervous system, DALYs = disability-adjusted life years.

The study revealed that forecasted age-standardized incidence rates (ASIR), age-standardized mortality, and DALYs for CNS cancer were slightly higher in males than in females, whereas the age-standardized prevalence rate (ASPR) was notably lower in males (Fig. 7). Globally, the ASIR, age-standardized mortality, and DALYs associated with CNS cancer are expected to continue their downward trends. For females, these standardized rate patterns mirror those seen in males, but the ASPR is projected to rise. Projections indicate that post-2040, age-standardized rates for CNS cancer will stabilize at relatively low levels, primarily due to advancements in therapeutic strategies (Fig. 7). Despite anticipated reductions in ASIR, mortality, and DALYs, the absolute numbers of new diagnoses, existing cases, and deaths are expected to increase over the next 25 years, influenced by demographic aging and relaxed population policies. The shaded regions in Figure 8 suggest that variations in new diagnoses, cases, deaths, and DALYs could be substantial if corresponding rates fluctuate by 1% annually, emphasizing the importance of preventive strategies and healthcare improvements. Under current projections, the number of new male cases is estimated to rise from 1,87,599 in 2021 to 2,18,260 in 2046, with prevalence increasing from 4,86,580 to 6,44,430 and deaths from 1,40,636 to 1,43,141. Conversely, DALYs are anticipated to decrease from 48,68,810 to 44,92,097 during the same period. For females, new diagnoses are projected to grow from 1,52,858 in 2021 to 1,85,775 in 2046, with prevalence rising from 4,62,800 to 6,55,342. Deaths among females are expected to increase from 1,03,467 in 2021 to 1,06,189 by 2046. However, DALYs are forecasted to decline from 36,74,077 to 35,56,833 over the same period.

In summary, total new CNS cancer cases are projected to increase from 3,38,558 in 2021 to 4,02,128 in 2046, with prevalence rising from 9,48,131 to 12,95,944. Mortality is expected to grow from 2,42,115 in 2021 to 2,47,735 in 2046, while DALYs are predicted to decline from 85,14,243 to 80,17,209 during this timeframe (Figs. 7 and 8).

## 4. Discussion

This investigation provides a comprehensive epidemiological analysis of global CNS cancer statistics, encompassing incidence, prevalence, mortality, and DALYs, derived from extensive data sources. The findings reveal an increasing global burden of CNS cancer, with prevalent cases more than doubling and deaths also increasing substantially over the past 3 decades. In 2021, there were an estimated 3,60,000 new cases and nearly 9,80,000 existing cases of CNS cancer worldwide.

Projections based on the BAPC model suggest that, in the absence of effective interventions, the total incidence for males could reach approximately 2,20,000 cases by 2040, with mortality estimated at 1,50,000. For females, the projected incidence is around 1,80,000 cases, with deaths anticipated at 1,10,000. Gender differences in the burden of CNS cancer are evident. Specifically,^[23]^ although CNS cancer incidence is generally higher in men than in women worldwide, this pattern is not consistent across all age groups. However, women may experience higher CNS cancer incidence or mortality in certain age groups. For example, our data show that women appear to be at greater risk as they age, perhaps because of age differences. This may be due to multiple factors, including biological sex differences, environmental exposures, lifestyle choices, and access to health care.^[24,25]^

The burden of CNS cancer varies across regions with differing levels of economic and social development.^[26]^ While the global burden has increased to varying degrees, mortality and DALY ratios are declining in more developed regions. This trend likely reflects enhanced resource allocation, advanced healthcare infrastructure, improved treatments, earlier and more accurate diagnoses, and higher educational levels, which collectively enable better patient engagement in treatment and prevention efforts. For example,^[27,28]^ Central Asia has seen a significant rise in CNS cancer burden, while East Asia has experienced a reduction due to improved prognosis and treatment in developed areas. Conversely, lower national development correlates with higher DALYs, reflecting limited access to specialized care. The highest number of deaths occurs in individuals over 60, particularly in Japan, while incidence rates and fatalities among children aged 1 to 10 years have declined significantly. This change may be due to increased awareness of pediatric tumors, advances in screening techniques, and more effective treatment options.^[29]^

Demographic aging exerts a greater influence in low and middle-SDI regions, while population growth primarily impacts high-middle-SDI regions. Trends in age-standardized DALYs suggest that epidemiological changes positively affect high and high-middle SDI regions, though the same shifts appear detrimental in low and middle SDI regions, highlighting disparities in progress against CNS cancer.^[30]^

Using GBD data, this analysis outlined temporal patterns in the CNS cancer burden over the past 3 decades and projected its trajectory in global projections over the next 25 years. The findings indicate consistent increases in ASIR, prevalence, and mortality from 1990 to 2021.^[28]^ Absolute numbers of new cases, existing cases, deaths, and DALYs have risen over the same period.

Projections suggest that, over the next quarter-century, ASIR, age-standardized mortality, and DALYs associated with CNS cancer will decline. However, the absolute numbers of new cases, prevalence, and fatalities are expected to continue rising globally, with the overall disease burden projected to increase substantially over the next 25 years.^[31]^ This trend is consistent across genders. This expected change reflects the influence of several factors. Although the age-standardized indicators are expected to improve in the next 25 years, the absolute burden of CNS cancer will continue to increase due to changes in population structure and socioeconomic development. This requires governments and all sectors of society to work together to adopt comprehensive prevention and control measures to effectively address this public health issue. Projected trends should be interpreted with caution. All forecasts are scenario-based and assume the continuation of historical patterns under the GBD modeling framework. Future trajectories may be altered by unforeseen changes in treatment effectiveness, diagnostic practices, health policies, or the distribution of risk factors, which are not explicitly accounted for in these projections.

A frontier analysis uncovered additional discrepancies among nations in tackling the burden of CNS cancer. Frontier analysis identifies the lowest observed burden achieved at comparable SDI levels and serves as a descriptive benchmark for cross-country comparison rather than a causal or normative standard.^[19]^ The observed combination of low recorded incidence and high DALY burden in some low-SDI regions should be interpreted cautiously. Limited access to neuroimaging, pathology services, and cancer registries may lead to underdiagnosis and underreporting of CNS cancer, resulting in underestimated incidence. At the same time, delayed diagnosis and restricted access to timely treatment may contribute to higher disability and premature mortality among detected cases, thereby inflating DALY estimates. These factors likely act in combination and highlight the influence of health system capacity and data quality on observed socioeconomic gradients. Nations with high SDI, such as Denmark, Norway, and Monaco, demonstrated a CNS cancer burden substantially above the frontier. However, the interpretation of frontier analysis requires caution, particularly in high-SDI settings. Greater distance from the frontier does not necessarily indicate suboptimal CNS cancer control, as higher age-standardized incidence may partly result from improved diagnostic capacity, more complete cancer registration, and longer survival. Accordingly, frontier gaps likely represent a combination of healthcare performance, diagnostic intensity, survival patterns, and data completeness rather than disease control alone.^[32]^ These findings may help identify settings where additional evaluation of diagnostic pathways, registry completeness, access to specialized care, and long-term survivorship patterns could be informative.

These adverse trends may present significant challenges for CNS cancer management and prevention efforts. If this escalation continues, CNS cancer could become a critical public health concern in regions currently experiencing a relatively lower disease burden. To address this growing challenge, national and regional health authorities must adopt proactive, multidisciplinary approaches across health, education, and economic sectors. Additionally, targeted strategies to improve diagnosis and treatment are essential to mitigating the increasing burden and reducing the impact of CNS cancer.

### 4.1. Research limitations

This study has several limitations. GBD 2021 estimates are subject to uncertainties related to data availability, quality, and modeling assumptions, particularly in countries with limited surveillance capacity, which may lead to the underestimation of within-country and cross-national disparities. Analyses conducted at the national or regional level cannot fully capture internal socioeconomic heterogeneity. In addition, CNS cancer comprises heterogeneous histological subtypes, and variation in subtype composition across socioeconomic settings may influence observed SDI-related patterns; however, subtype-specific analyses and projections were not feasible due to limitations in the granularity and consistency of current GBD data. Furthermore, the BAPC-based forecasts should be interpreted as exploratory, scenario-based projections rather than validated predictions. Because no formal out-of-sample validation or additional diagnostic assessment was performed, these projections are intended to illustrate the possible continuation of historical patterns under the GBD framework and should not be interpreted as deterministic estimates of future burden.

## 5. Conclusions

CNS cancer remains a significant global health challenge. From 1990 to 2021, age-standardized incidence and prevalence increased, while the age-standardized DALY rate declined modestly. Substantial variation across SDI settings and frontier gaps suggests that the observed burden is shaped not only by disease occurrence but also by differences in diagnosis, registration, survival, and health system capacity. Therefore, these findings should be interpreted as descriptive evidence of cross-national heterogeneity rather than direct evidence of suboptimal control in higher-SDI settings. Projections further suggest that absolute numbers of cases, deaths, and prevalent cases may continue to rise over the next 25 years, underscoring the need for continued investment in surveillance, access to care, and health system planning.

## Acknowledgments

We extend our gratitude to the JD_GBDR Study Groups for their contributions to the GBD database.

## Author contributions

**Conceptualization:** Jing Wang, Lei Zhang, Yanjun Wang.

**Data curation:** Jing Wang, Lei Zhang, Yanjun Wang, Yinfu Xu.

**Formal analysis:** Jing Wang, Lei Zhang, Yanjun Wang, Yinfu Xu.

**Investigation:** Jing Wang, Jie Liu.

**Methodology:** Jing Wang, Lei Zhang, Jie Liu, Yinfu Xu.

**Resources:** Yinfu Xu.

**Visualization:** Yuchen Guo.

**Writing – original draft:** Jing Wang, Lei Zhang, Peijun Han.















## References

[1] Louis DN Perry A Reifenberger G. The 2016 World Health Organization classification of tumors of the central nervous system: a summary. Acta Neuropathol. 2016;131:803–20. 27157931 10.1007/s00401-016-1545-1

[2] Leece R Xu J Ostrom QT Chen Y Kruchko C Barnholtz-Sloan JS. Global incidence of malignant brain and other central nervous system tumors by histology, 2003-2007. Neuro Oncol. 2017;19:1553–64. 28482030 10.1093/neuonc/nox091 PMC5737839

[3] Fitzmaurice C Allen C Barber RM; Global Burden of Disease Cancer Collaboration. Global, regional, and national cancer incidence, mortality, years of life lost, years lived with disability, and disability-adjusted life-years for 32 cancer groups, 1990 to 2015: a systematic analysis for the global burden of disease study. JAMA Oncol. 2017;3:524–48. 27918777 10.1001/jamaoncol.2016.5688 PMC6103527

[4] GBD 2016 Disease and Injury Incidence and Prevalence Collaborators. Global, regional, and national incidence, prevalence, and years lived with disability for 328 diseases and injuries for 195 countries, 1990–2016: a systematic analysis for the Global Burden of Disease Study 2016. Lancet. 2017;390:1211–59. 28919117 10.1016/S0140-6736(17)32154-2 PMC5605509

[5] GBD 2016 Mortality Collaborators. Global, regional, and national under-5 mortality, adult mortality, age-specific mortality, and life expectancy, 1970–2016: a systematic analysis for the Global Burden of Disease Study 2016. Lancet. 2017;390:1084–150. 28919115 10.1016/S0140-6736(17)31833-0 PMC5605514

[6] Davis FG McCarthy BJ Freels S Kupelian V Bondy ML. The conditional probability of survival of patients with primary malignant brain tumors: surveillance, epidemiology, and end results (SEER) data. Cancer. 1999;85:485–91. 10023719

[7] GBD 2016 Causes of Death Collaborators. Global, regional, and national age-sex specific mortality for 264 causes of death, 1980–2016: a systematic analysis for the Global Burden of Disease Study 2016. Lancet. 2017;390:1151–210. 28919116 10.1016/S0140-6736(17)32152-9 PMC5605883

[8] GBD 2019 Risk Factors Collaborators. Global burden of 87 risk factors in 204 countries and territories, 1990–2019: a systematic analysis for the Global Burden of Disease Study 2019. Lancet. 2020;396:1223–49. 33069327 10.1016/S0140-6736(20)30752-2 PMC7566194

[9] GBD 2016 DALYs and HALE Collaborators. Global, regional, and national disability-adjusted life-years (DALYs) for 333 diseases and injuries and healthy life expectancy (HALE) for 195 countries and territories, 1990–2016: a systematic analysis for the Global Burden of Disease Study 2016. Lancet. 2017;390:1260–344. 28919118 10.1016/S0140-6736(17)32130-X PMC5605707

[10] Fitzmaurice C Akinyemiju TF Al Lami FH. Global, regional, and national cancer incidence, mortality, years of life lost, years lived with disability, and disability-adjusted life-years for 29 cancer groups, 1990 to 2016: a systematic analysis for the global burden of disease study. JAMA Oncol. 2018;4:1553–68. 29860482 10.1001/jamaoncol.2018.2706 PMC6248091

[11] GBD 2021 Diseases and Injuries Collaborators. Global incidence, prevalence, years lived with disability (YLDs), disability-adjusted life-years (DALYs), and healthy life expectancy (HALE) for 371 diseases and injuries in 204 countries and territories and 811 subnational locations, 1990–2021: a systematic analysis for the Global Burden of Disease Study 2021. Lancet. 2024;403:2133–61. 38642570 10.1016/S0140-6736(24)00757-8 PMC11122111

[12] GBD 2021 Diabetes Collaborators. Global, regional, and national burden of diabetes from 1990 to 2021, with projections of prevalence to 2050: a systematic analysis for the Global Burden of Disease Study 2021. Lancet. 2023;402:203–34. 37356446 10.1016/S0140-6736(23)01301-6 PMC10364581

[13] GBD 2021 Low Back Pain Collaborators. Global, regional, and national burden of low back pain, 1990–2020, its attributable risk factors, and projections to 2050: a systematic analysis of the Global Burden of Disease Study 2021. Lancet Rheumatol. 2023;5:e316–29. 37273833 10.1016/S2665-9913(23)00098-X PMC10234592

[14] GBD 2019 Diseases and Injuries Collaborators. Global burden of 369 diseases and injuries in 204 countries and territories, 1990–2019: a systematic analysis for the Global Burden of Disease Study 2019. Lancet. 2020;396:1204–22. 33069326 10.1016/S0140-6736(20)30925-9 PMC7567026

[15] Yang X Zhang T Zhang H Sang S Chen H Zuo X. Temporal trend of gastric cancer burden along with its risk factors in China from 1990 to 2019, and projections until 2030: comparison with Japan, South Korea, and Mongolia. Biomark Res. 2021;9:84. 34784961 10.1186/s40364-021-00340-6 PMC8597246

[16] Xie J Wang M Long Z. Global burden of type 2 diabetes in adolescents and young adults, 1990-2019: systematic analysis of the Global Burden of Disease Study 2019. BMJ. 2022;379:e072385. 36740855 10.1136/bmj-2022-072385 PMC9727920

[17] GBD 2015 Mortality and Causes of Death Collaborators. Global, regional, and national life expectancy, all-cause mortality, and cause-specific mortality for 249 causes of death, 1980–2015: a systematic analysis for the Global Burden of Disease Study 2015. Lancet. 2016;388:1459–544. 27733281 10.1016/S0140-6736(16)31012-1 PMC5388903

[18] Zhou L Deng Y Li N. Global, regional, and national burden of Hodgkin lymphoma from 1990 to 2017: estimates from the 2017 Global Burden of Disease study. J Hematol Oncol. 2019;12:107. 31640759 10.1186/s13045-019-0799-1 PMC6805485

[19] Mohammadi E Ghasemi E Azadnajafabad S. A global, regional, and national survey on burden and Quality of Care Index (QCI) of brain and other central nervous system cancers; global burden of disease systematic analysis 1990-2017. PLoS One. 2021;16:e0247120. 33617563 10.1371/journal.pone.0247120 PMC7899371

[20] Hankey BF Ries LA Kosary CL. Partitioning linear trends in age-adjusted rates. Cancer Causes Control. 2000;11:31–5. 10680727 10.1023/a:1008953201688

[21] Møller B Fekjaer H Hakulinen T. Prediction of cancer incidence in the Nordic countries: empirical comparison of different approaches. Stat Med. 2003;22:2751–66. 12939784 10.1002/sim.1481

[22] Riebler A Held L. Projecting the future burden of cancer: Bayesian age-period-cohort analysis with integrated nested Laplace approximations. Biom J. 2017;59:531–49. 28139001 10.1002/bimj.201500263

[23] Huang J Chan SC Lok V; NCD Global Health Research Group. Disease burden, risk factors, and trends of primary central nervous system (CNS) cancer: a global study of registries data. Neuro Oncol. 2023;25:995–1005. 36048182 10.1093/neuonc/noac213 PMC10158137

[24] Rubin JB Lagas JS Broestl L. Sex differences in cancer mechanisms. Biol Sex Differ. 2020;11:17. 32295632 10.1186/s13293-020-00291-x PMC7161126

[25] Azadnajafabad S Moghaddam SS Mohammadi E. Global, regional, and national burden and quality of care index (QCI) of thyroid cancer: a systematic analysis of the Global Burden of Disease Study 1990-2017. Cancer Med. 2021;10:2496–508. 33665966 10.1002/cam4.3823 PMC7982631

[26] GBD 2015 Neurological Disorders Collaborator Group. Global, regional, and national burden of neurological disorders during 1990–2015: a systematic analysis for the Global Burden of Disease Study 2015. Lancet Neurol. 2017;16:877–97. 28931491 10.1016/S1474-4422(17)30299-5 PMC5641502

[27] GBD 2016 Brain and Other CNS Cancer Collaborators. Global, regional, and national burden of brain and other CNS cancer, 1990–2016: a systematic analysis for the Global Burden of Disease Study 2016. Lancet Neurol. 2019;18:376–93. 30797715 10.1016/S1474-4422(18)30468-X PMC6416167

[28] Huang J Li H Yan H Li FX Tang M Lu DL. The comparative burden of brain and central nervous system cancers from 1990 to 2019 between China and the United States and predicting the future burden. Front Public Health. 2022;10:1018836. 36339132 10.3389/fpubh.2022.1018836 PMC9635888

[29] Fitzmaurice C Abate D Abbasi N; Global Burden of Disease Cancer Collaboration. Global, regional, and national cancer incidence, mortality, years of life lost, years lived with disability, and disability-adjusted life-years for 29 cancer groups, 1990 to 2017: a systematic analysis for the global burden of disease study. JAMA Oncol. 2019;5:1749–68. 31560378 10.1001/jamaoncol.2019.2996 PMC6777271

[30] Zhu J Li S Li X Wang L Du L Qiu Y. Impact of population ageing on cancer-related disability-adjusted life years: a global decomposition analysis. J Glob Health. 2024;14:04144. 39024622 10.7189/jogh.14.04144 PMC11259023

[31] Liu X Cheng LC Gao TY Luo J Zhang C. The burden of brain and central nervous system cancers in Asia from 1990 to 2019 and its predicted level in the next twenty-five years: burden and prediction model of CNS cancers in Asia. BMC Public Health. 2023;23:2522. 38104107 10.1186/s12889-023-17467-w PMC10724911

[32] GBD 2015 Healthcare Access and Quality Collaborators. Healthcare Access and Quality Index based on mortality from causes amenable to personal health care in 195 countries and territories, 1990–2015: a novel analysis from the Global Burden of Disease Study 2015. Lancet. 2017;390:231–66. 28528753 10.1016/S0140-6736(17)30818-8 PMC5528124

