# Supplementary material for: Global, regional, and national analysis of CNS cancers burden from 1990 to 2021 and forecast of disease trend over the next 25 years
Source: Medicine (Baltimore). 2026 Aug 14;105(33):e50073. doi: 10.1097/MD.0000000000050073 (PMC13480817; doi:10.1097/MD.0000000000050073)
Supplement: Supplementary file 1 [file medi-105-e50073-s001.docx]

Table S1 Global prevalence, incidence, deaths, and DALYs of CNS cancer by age group and their rates

| Age | Incidence | | Prevalence | | Deaths | | DALYs | |
| --- | --- | --- | --- | --- | --- | --- | --- | --- |
|  | Number | Rate | Number | Rate | Number | Rate | Number | Rate |
| <5 | 11617.27(8947.40,14807.47) | 1.77(1.36,2.25) | 59475.35(46056.85,75035.33) | 9.04(7.00,11.40) | 5660.40(4242.32,7404.99) | 0.86(0.64,1.13) | 502601.05(376858.94,656922.69) | 76.36(57.26,99.81) |
| 5 to 9 | 11509.22(9336.10,13926.50) | 1.68(1.36,2.03) | 53660.73(44497.56,64793.64) | 7.81(6.48,9.43) | 5862.96(4608.01,7129.83) | 0.85(0.67,1.04) | 490209.74(385233.37,595774.57) | 71.35(56.07,86.71) |
| 10 to 14 | 9964.59(8443.78,11967.15) | 1.49(1.27,1.80) | 49743.07(42589.06,60066.95) | 7.46(6.39,9.01) | 4832.50(3977.72,5808.12) | 0.72(0.60,0.87) | 378498.53(311973.10,454289.84) | 56.78(46.80,68.15) |
| 15 to 19 | 7443.44(6137.85,8729.33) | 1.19(0.98,1.40) | 36334.48(30180.39,42724.40) | 5.82(4.84,6.85) | 3566.65(2924.05,4256.33) | 0.57(0.47,0.68) | 261630.36(214433.70,312229.09) | 41.93(34.37,50.04) |
| 20 to 24 | 8465.52(7153.60,9690.28) | 1.42(1.20,1.62) | 42967.64(36538.38,48757.78) | 7.20(6.12,8.16) | 3800.44(3139.02,4457.43) | 0.64(0.53,0.75) | 260719.50(215174.46,304638.76) | 43.66(36.03,51.01) |
| 25 to 29 | 10726.65(9251.55,12136.53) | 1.82(1.57,2.06) | 56210.37(48677.65,63535.98) | 9.55(8.27,10.80) | 4940.90(4131.67,5777.72) | 0.84(0.70,0.98) | 314494.55(262166.19,367350.63) | 53.45(44.56,62.44) |
| 30 to 34 | 14620.73(12217.94,17271.90) | 2.42(2.02,2.86) | 70643.79(59656.83,82595.43) | 11.69(9.87,13.66) | 7201.81(5967.14,8740.15) | 1.19(0.99,1.45) | 421833.39(349613.13,511628.93) | 69.78(57.84,84.64) |
| 35 to 39 | 16388.81(14240.97,19088.76) | 2.92(2.54,3.40) | 65613.84(57670.54,76257.04) | 11.70(10.28,13.60) | 9079.97(7735.92,10695.47) | 1.62(1.38,1.91) | 485972.00(413969.59,572424.95) | 86.65(73.81,102.06) |
| 40 to 44 | 18041.71(15310.54,21473.95) | 3.61(3.06,4.29) | 55778.50(48618.26,65495.84) | 11.15(9.72,13.09) | 11544.61(9719.52,13823.57) | 2.31(1.94,2.76) | 558910.54(470333.23,668890.96) | 111.73(94.02,133.71) |
| 45 to 49 | 21768.35(18695.43,25538.55) | 4.60(3.95,5.39) | 61500.05(54191.68,71571.14) | 12.99(11.44,15.12) | 14714.24(12513.76,17410.64) | 3.11(2.64,3.68) | 639615.45(544594.13,756513.00) | 135.08(115.01,159.77) |
| 50 to 54 | 28134.31(24172.93,33129.70) | 6.32(5.43,7.45) | 68673.94(60147.34,79257.06) | 15.44(13.52,17.81) | 20352.12(17458.19,24160.23) | 4.57(3.92,5.43) | 786776.96(673764.48,933066.64) | 176.83(151.43,209.71) |
| 55 to 59 | 34157.12(29608.82,39536.18) | 8.63(7.48,9.99) | 74524.74(65831.91,84361.01) | 18.83(16.64,21.32) | 26565.75(22889.29,30866.85) | 6.71(5.78,7.80) | 902010.95(775445.75,1048260.83) | 227.94(195.95,264.89) |
| 60 to 64 | 34544.49(30571.69,39160.84) | 10.79(9.55,12.24) | 64341.97(57606.85,71135.04) | 20.10(18.00,22.23) | 28506.71(25122.85,32648.02) | 8.91(7.85,10.20) | 834032.72(737282.09,954396.72) | 260.60(230.37,298.20) |
| 65 to 69 | 37658.81(32709.56,42616.30) | 13.65(11.86,15.45) | 63323.30(56408.98,70668.03) | 22.96(20.45,25.62) | 32248.17(28024.09,36539.61) | 11.69(10.16,13.25) | 795479.62(691114.94,903216.66) | 288.38(250.55,327.44) |
| 70 to 74 | 33966.06(30066.26,37879.21) | 16.50(14.61,18.40) | 57216.24(50364.25,62296.60) | 27.80(24.47,30.26) | 29594.77(26167.05,33153.28) | 14.38(12.71,16.11) | 601713.51(531906.48,676128.17) | 292.32(258.41,328.47) |
| 75 to 79 | 24567.70(21003.96,27251.80) | 18.63(15.93,20.66) | 37082.87(32054.57,40674.35) | 28.12(24.31,30.84) | 21849.07(18737.91,24250.84) | 16.57(14.21,18.39) | 355394.07(304945.15,395431.75) | 269.47(231.22,299.83) |
| 80 to 84 | 17187.91(14235.50,19158.15) | 19.62(16.25,21.87) | 27217.40(21630.91,31114.03) | 31.08(24.70,35.53) | 15356.47(12940.42,17056.32) | 17.53(14.78,19.47) | 195895.67(165056.11,217975.97) | 223.67(188.46,248.88) |
| 85 to 89 | 10247.73(8416.36,11622.69) | 22.41(18.41,25.42) | 18398.23(14144.60,22236.56) | 40.24(30.94,48.63) | 8653.86(7152.12,9731.15) | 18.93(15.64,21.28) | 88418.33(73265.12,99591.22) | 193.38(160.24,217.82) |
| 90 to 94 | 5142.02(4008.60,5921.68) | 28.74(22.41,33.10) | 11592.27(8457.98,14109.26) | 64.80(47.28,78.87) | 3325.32(2640.42,3763.61) | 18.59(14.76,21.04) | 30233.09(24069.88,34245.29) | 169.00(134.55,191.43) |
| 95 plus | 1329.84(975.88,1552.28) | 24.40(17.91,28.48) | 980.36(699.82,1147.57) | 17.99(12.84,21.06) | 970.07(723.28,1116.43) | 17.80(13.27,20.48) | 8155.18(6090.27,9392.19) | 149.63(111.74,172.32) |
