# Supplementary material for: Global, regional, and national analysis of CNS cancers burden from 1990 to 2021 and forecast of disease trend over the next 25 years
Source: Medicine (Baltimore). 2026 Aug 14;105(33):e50073. doi: 10.1097/MD.0000000000050073 (PMC13480817; doi:10.1097/MD.0000000000050073)
Supplement: Supplementary file 2 [file medi-105-e50073-s003.docx]

Table S2	Prevalence, incidence, deaths, and DALYs and their age-standardized rates in female CNS cancer patients worldwide, between 1990 and 2021

| Year | Incidence | | Prevalence | | Deaths | | DALYs | |
| --- | --- | --- | --- | --- | --- | --- | --- | --- |
|  | Number | Rate | Number | Rate | Number | Rate | Number | Rate |
| 1990 | 79537(67859,92584) | 3.36(2.91,3.85) | 206798(175555,242220) | 8.21(7.06,9.47) | 60841(51618,71371) | 2.63(2.26,3.03) | 2585503(2058074,3293795) | 103.63(83.99,128.70) |
| 1991 | 82025(70372,94466) | 3.40(2.96,3.87) | 214455(182505,247962) | 8.37(7.20,9.55) | 62481(53538,72585) | 2.65(2.30,3.04) | 2644016(2122815,3300808) | 104.31(85.27,127.43) |
| 1992 | 84114(72185,97256) | 3.43(2.97,3.91) | 220396(187515,253172) | 8.47(7.29,9.62) | 63928(54637,74562) | 2.66(2.31,3.05) | 2689604(2167153,3328229) | 104.52(85.49,127.68) |
| 1993 | 86021(74147,97696) | 3.45(3.00,3.87) | 225346(192034,255836) | 8.53(7.34,9.58) | 65231(56202,74570) | 2.67(2.32,3.02) | 2725266(2217863,3321669) | 104.41(86.13,125.32) |
| 1994 | 88430(75742,100411) | 3.49(3.02,3.92) | 232537(198215,260499) | 8.69(7.47,9.69) | 66694(57085,76212) | 2.68(2.32,3.03) | 2764581(2234940,3337749) | 104.50(85.54,124.83) |
| 1995 | 90783(78280,102132) | 3.52(3.06,3.94) | 239568(205371,269429) | 8.84(7.63,9.86) | 68055(58458,77291) | 2.69(2.33,3.04) | 2800016(2288458,3401876) | 104.44(86.13,125.08) |
| 1996 | 93347(80868,104176) | 3.56(3.11,3.95) | 247783(213546,275429) | 9.01(7.83,9.96) | 69525(60012,78262) | 2.71(2.35,3.01) | 2837209(2325076,3404439) | 104.39(86.33,123.59) |
| 1997 | 95679(83282,105649) | 3.59(3.14,3.94) | 254927(219850,281057) | 9.14(7.92,10.05) | 71021(61814,79030) | 2.72(2.38,3.01) | 2871509(2374825,3389301) | 104.17(86.80,121.87) |
| 1998 | 97711(84709,107891) | 3.61(3.14,3.96) | 260998(224660,286067) | 9.22(7.96,10.06) | 72517(62795,80824) | 2.72(2.37,3.02) | 2900768(2394517,3419443) | 103.76(86.27,121.44) |
| 1999 | 100008(86852,109438) | 3.63(3.17,3.95) | 269933(233779,295387) | 9.41(8.17,10.26) | 74130(64319,81857) | 2.74(2.39,3.01) | 2935519(2433205,3428556) | 103.50(86.24,120.10) |
| 2000 | 102643(89481,111754) | 3.66(3.21,3.97) | 282127(245335,307068) | 9.69(8.46,10.53) | 76036(65994,83560) | 2.75(2.40,3.02) | 2983800(2483866,3465334) | 103.68(86.63,119.75) |
| 2001 | 104613(92182,114285) | 3.66(3.23,3.99) | 292358(258143,316478) | 9.89(8.76,10.68) | 77416(67774,85306) | 2.75(2.41,3.02) | 3014157(2552923,3474005) | 103.11(87.57,118.39) |
| 2002 | 106731(93187,117012) | 3.67(3.21,4.02) | 299619(262831,326013) | 9.98(8.77,10.85) | 78959(68726,87252) | 2.75(2.40,3.04) | 3042728(2565744,3504001) | 102.45(86.57,117.73) |
| 2003 | 108531(95198,118441) | 3.66(3.21,3.99) | 304542(270175,330122) | 9.98(8.86,10.79) | 80176(69711,87850) | 2.74(2.39,2.99) | 3061963(2601606,3510808) | 101.40(86.19,116.09) |
| 2004 | 111066(98020,121628) | 3.67(3.24,4.02) | 311785(277892,337939) | 10.05(8.95,10.89) | 81654(71846,89974) | 2.73(2.40,3.00) | 3092754(2650586,3517547) | 100.69(86.35,114.46) |
| 2005 | 114669(102602,125061) | 3.71(3.32,4.05) | 323206(290438,350448) | 10.24(9.22,11.10) | 83571(74278,91558) | 2.73(2.43,2.99) | 3142437(2733404,3571110) | 100.54(87.60,114.25) |
| 2006 | 117835(105148,127569) | 3.74(3.33,4.04) | 334448(300339,360136) | 10.41(9.36,11.21) | 85039(75552,92439) | 2.72(2.42,2.96) | 3176325(2763715,3568904) | 99.80(86.95,112.14) |
| 2007 | 120780(107775,130667) | 3.75(3.34,4.05) | 344095(311051,371794) | 10.52(9.51,11.36) | 86547(76589,93775) | 2.71(2.39,2.93) | 3206457(2808252,3576180) | 98.90(86.55,110.43) |
| 2008 | 124386(111085,135908) | 3.78(3.37,4.13) | 356025(321646,390520) | 10.69(9.65,11.72) | 88654(78943,97056) | 2.71(2.41,2.97) | 3263241(2857955,3669036) | 98.79(86.48,111.12) |
| 2009 | 127453(113715,139544) | 3.79(3.38,4.15) | 366440(330389,401446) | 10.81(9.75,11.85) | 90503(80065,99208) | 2.70(2.39,2.96) | 3306708(2883499,3711214) | 98.23(85.72,110.30) |
| 2010 | 130298(117121,141340) | 3.79(3.41,4.11) | 375858(341764,408198) | 10.89(9.90,11.84) | 92243(82186,100037) | 2.69(2.40,2.92) | 3346067(2942871,3695181) | 97.56(85.65,108.13) |
| 2011 | 133452(119307,145746) | 3.80(3.40,4.15) | 387098(351926,420673) | 11.03(10.02,12.01) | 94006(83073,102100) | 2.68(2.37,2.91) | 3385320(2976258,3752586) | 96.92(85.22,107.75) |
| 2012 | 137109(123505,151948) | 3.82(3.44,4.24) | 398238(364668,444023) | 11.15(10.20,12.44) | 96303(85733,106233) | 2.68(2.39,2.96) | 3442145(3055265,3852499) | 96.73(85.82,108.27) |
| 2013 | 140576(125119,155900) | 3.84(3.42,4.26) | 409277(371328,456315) | 11.26(10.23,12.57) | 98376(87254,108522) | 2.68(2.37,2.95) | 3499318(3109436,3918506) | 96.53(85.63,108.24) |
| 2014 | 144491(130678,157413) | 3.86(3.49,4.22) | 421660(388399,465439) | 11.40(10.51,12.61) | 100851(90026,108687) | 2.68(2.39,2.89) | 3563677(3187435,3934658) | 96.50(86.06,106.66) |
| 2015 | 148531(133975,161563) | 3.88(3.50,4.23) | 434169(399124,477078) | 11.55(10.64,12.70) | 103600(92134,112285) | 2.69(2.39,2.92) | 3635769(3233951,4006856) | 96.65(85.82,106.58) |
| 2016 | 152576(135316,167008) | 3.91(3.47,4.28) | 447548(406868,495032) | 11.71(10.64,12.95) | 106201(92879,116110) | 2.69(2.36,2.94) | 3705299(3258408,4089951) | 96.72(84.98,106.73) |
| 2017 | 156570(137989,172500) | 3.93(3.46,4.33) | 461206(412616,511972) | 11.87(10.63,13.19) | 108563(94841,119465) | 2.69(2.35,2.96) | 3765924(3287452,4191676) | 96.57(84.25,107.66) |
| 2018 | 160451(139020,175673) | 3.94(3.43,4.32) | 473367(417650,526226) | 12.00(10.61,13.34) | 110901(96237,120957) | 2.68(2.33,2.93) | 3823513(3324603,4187198) | 96.36(83.74,105.60) |
| 2019 | 163540(143393,182379) | 3.94(3.45,4.40) | 483260(430563,541391) | 12.07(10.80,13.54) | 112701(98535,124730) | 2.67(2.34,2.96) | 3863129(3456228,4366692) | 95.75(85.33,108.26) |
| 2020 | 164222(143829,180976) | 3.87(3.40,4.27) | 479930(426949,536985) | 11.80(10.51,13.29) | 113605(99008,123875) | 2.63(2.29,2.87) | 3855241(3373118,4248142) | 93.91(82.10,103.89) |
| 2021 | 167353(147253,187695) | 3.88(3.43,4.35) | 485235(432259,548646) | 11.76(10.48,13.28) | 115723(102256,128972) | 2.62(2.31,2.92) | 3897852(3458116,4340528) | 93.46(82.86,104.06) |
