# Supplementary material for: Global, regional, and national analysis of CNS cancers burden from 1990 to 2021 and forecast of disease trend over the next 25 years
Source: Medicine (Baltimore). 2026 Aug 14;105(33):e50073. doi: 10.1097/MD.0000000000050073 (PMC13480817; doi:10.1097/MD.0000000000050073)
Supplement: Supplementary file 3 [file medi-105-e50073-s007.docx]

Table S3 Incidence, prevalence, deaths, and DALYs and their age-standardized rates in male CNS cancer patients worldwide, between 1990 and 2021

| Year | Incidence | | Prevalence | | Deaths | | DALYs | |
| --- | --- | --- | --- | --- | --- | --- | --- | --- |
|  | Number | Rate | Number | Rate | Number | Rate | Number | Rate |
| 1990 | 93549(73313,114995) | 4.18(3.32,5.05) | 227626(182823,271052) | 9.13(7.45,10.90) | 75378(57815,93249) | 3.49(2.73,4.28) | 3372978(2497272,4250999) | 136.51(102.54,172.09) |
| 1991 | 96200(75856,117281) | 4.21(3.36,5.07) | 234621(188624,276537) | 9.25(7.62,10.82) | 79258(61750,97881) | 3.53(2.80,4.32) | 3448146(2543204,4290939) | 137.20(103.79,170.63) |
| 1992 | 98743(77686,120467) | 4.24(3.42,5.14) | 241160(192989,285993) | 9.35(7.64,10.99) | 83164(65376,102901) | 3.58(2.85,4.39) | 3515889(2593111,4408154) | 137.67(103.44,171.22) |
| 1993 | 101221(79799,123314) | 4.27(3.41,5.12) | 247065(200012,296889) | 9.43(7.78,11.29) | 88660(69126,108018) | 3.63(2.87,4.37) | 3575568(2682940,4559557) | 137.96(104.86,174.66) |
| 1994 | 104128(83113,127447) | 4.32(3.50,5.24) | 254269(204602,300391) | 9.57(7.84,11.29) | 85103(66947,103734) | 3.60(2.86,4.34) | 3741999(2828743,4702230) | 138.67(106.29,173.56) |
| 1995 | 107074(85681,128960) | 4.38(3.53,5.23) | 262068(213595,310242) | 9.74(8.06,11.39) | 77360(59545,95255) | 3.51(2.76,4.28) | 3693864(2789282,4585074) | 138.76(106.62,171.00) |
| 1996 | 109663(87992,132716) | 4.41(3.57,5.28) | 268835(220215,318533) | 9.85(8.13,11.59) | 90474(71065,110625) | 3.64(2.89,4.40) | 3637477(2705666,4567393) | 138.44(105.36,172.76) |
| 1997 | 112113(89084,134891) | 4.44(3.57,5.31) | 274726(223474,323893) | 9.92(8.17,11.54) | 99653(79042,118012) | 3.67(2.92,4.31) | 3992214(3081254,4873243) | 136.19(105.59,165.35) |
| 1998 | 114464(91664,137821) | 4.46(3.60,5.34) | 281041(228959,333913) | 10.02(8.27,11.78) | 94784(74743,113943) | 3.69(2.93,4.43) | 4015919(3174567,4927412) | 134.90(106.93,164.52) |
| 1999 | 116806(93355,139724) | 4.48(3.60,5.32) | 289290(236291,342820) | 10.18(8.40,11.93) | 96662(76192,115685) | 3.70(2.93,4.39) | 3790023(2855260,4657462) | 138.55(105.64,168.69) |
| 2000 | 120093(96269,142909) | 4.54(3.65,5.36) | 301658(246606,354706) | 10.49(8.66,12.25) | 110047(87626,132852) | 3.64(2.92,4.37) | 3976045(3056333,4876307) | 137.67(106.47,168.15) |
| 2001 | 122511(98059,145313) | 4.55(3.66,5.36) | 311597(257066,365019) | 10.69(8.86,12.44) | 125319(100376,151931) | 3.63(2.92,4.35) | 3830711(2901445,4770499) | 138.20(105.93,171.15) |
| 2002 | 124430(99422,147034) | 4.53(3.64,5.32) | 318052(263195,371034) | 10.76(8.95,12.47) | 101801(80755,122379) | 3.67(2.92,4.39) | 3934073(3011041,4822270) | 138.19(107.25,168.54) |
| 2003 | 126472(101917,148662) | 4.52(3.67,5.29) | 322471(268892,379643) | 10.75(9.00,12.57) | 122356(97041,146946) | 3.62(2.89,4.33) | 4265087(3320376,5302562) | 131.45(102.54,162.55) |
| 2004 | 129654(104730,154262) | 4.55(3.68,5.38) | 330473(275350,389028) | 10.84(9.09,12.68) | 103834(82617,125866) | 3.67(2.94,4.42) | 3867507(2950955,4798700) | 137.68(105.85,168.84) |
| 2005 | 133088(107364,159418) | 4.58(3.72,5.45) | 339480(280678,403058) | 10.97(9.13,12.91) | 92248(72210,111770) | 3.65(2.88,4.39) | 4330281(3367321,5395124) | 131.07(102.27,162.65) |
| 2006 | 135795(109237,160651) | 4.58(3.72,5.38) | 347697(286537,409658) | 11.05(9.17,12.92) | 137188(109730,167063) | 3.62(2.90,4.39) | 4183893(3249434,5105074) | 131.27(102.41,159.44) |
| 2007 | 139016(111754,164494) | 4.59(3.72,5.40) | 356432(293476,418508) | 11.13(9.21,12.99) | 105166(83572,125621) | 3.64(2.91,4.32) | 4140256(3213039,5105988) | 132.24(103.00,162.30) |
| 2008 | 143437(115324,172092) | 4.64(3.76,5.54) | 369090(303673,441738) | 11.34(9.37,13.48) | 128563(102363,154664) | 3.64(2.90,4.35) | 4068367(3181735,5018843) | 134.44(105.62,165.27) |
| 2009 | 147200(118487,177299) | 4.67(3.77,5.58) | 380283(312700,454907) | 11.48(9.48,13.67) | 107121(85139,127433) | 3.62(2.90,4.28) | 4116637(3205612,5069686) | 133.81(104.51,163.76) |
| 2010 | 150884(121343,180177) | 4.69(3.79,5.56) | 390923(320571,465889) | 11.61(9.56,13.77) | 112559(89303,135773) | 3.65(2.90,4.37) | 4736864(3690025,5857631) | 128.64(100.31,158.80) |
| 2011 | 154300(125436,186011) | 4.70(3.83,5.62) | 401410(330587,479256) | 11.74(9.70,13.94) | 115066(91797,137596) | 3.65(2.92,4.33) | 4648968(3634390,5812393) | 128.59(100.65,160.42) |
| 2012 | 158434(128685,191266) | 4.73(3.86,5.67) | 411919(338573,490537) | 11.86(9.77,14.07) | 117272(94504,142065) | 3.63(2.93,4.37) | 4568172(3545300,5601738) | 128.70(100.06,157.39) |
| 2013 | 161706(129495,193482) | 4.72(3.80,5.61) | 421344(343610,498578) | 11.93(9.77,14.05) | 120186(96656,145569) | 3.64(2.94,4.39) | 4390986(3445404,5426389) | 130.55(102.73,160.83) |
| 2014 | 166191(134003,199858) | 4.75(3.84,5.66) | 435299(354324,517368) | 12.12(9.90,14.34) | 131414(104655,158524) | 3.63(2.90,4.35) | 5009157(3859079,6313195) | 126.70(97.41,159.51) |
| 2015 | 170523(136380,203023) | 4.78(3.83,5.67) | 446897(361527,531168) | 12.25(9.94,14.50) | 134123(106227,162070) | 3.62(2.87,4.35) | 4441461(3496693,5487134) | 129.75(102.42,159.77) |
| 2016 | 174495(139698,209237) | 4.78(3.83,5.70) | 457939(370635,538315) | 12.35(10.04,14.46) | 140114(110691,171562) | 3.61(2.86,4.41) | 4513880(3534506,5601302) | 129.54(101.64,160.24) |
| 2017 | 178630(142007,215477) | 4.79(3.82,5.76) | 470197(377172,565043) | 12.48(10.03,14.96) | 98097(76861,116578) | 3.68(2.90,4.34) | 4805648(3738440,5916591) | 128.13(99.74,157.49) |
| 2018 | 183048(146235,222114) | 4.81(3.85,5.82) | 482009(386107,582844) | 12.59(10.11,15.17) | 86834(68484,106511) | 3.61(2.89,4.36) | 4981997(3759735,6303868) | 123.95(93.41,156.71) |
| 2019 | 187099(148834,228705) | 4.82(3.84,5.87) | 491826(389397,590251) | 12.66(10.04,15.17) | 81126(62609,99319) | 3.55(2.81,4.30) | 4875230(3764302,6036172) | 127.67(98.55,157.87) |
| 2020 | 187572(146602,231155) | 4.74(3.71,5.81) | 486926(381076,595744) | 12.35(9.68,15.10) | 141047(110155,173986) | 3.56(2.78,4.36) | 4943750(3860102,6207980) | 127.20(99.20,159.58) |
| 2021 | 190130(148118,232469) | 4.72(3.68,5.76) | 490044(387339,600066) | 12.28(9.72,15.05) | 142904(110662,174945) | 3.54(2.75,4.32) | 5014743(3827506,6403194) | 122.96(93.65,157.33) |
